# Amenamevir Transfer into Breast Milk in Patients with Herpes Simplex Virus Infection: A Brief Report of a Prospective Multicenter Study

**DOI:** 10.1111/1346-8138.70474

**Published:** 2026-08-31

**Authors:** Sachi Koinuma, Mikako Goto, Jumpei Saito, Hiroaki Aoki, Satoshi Hayashi, Tomoyoshi Takeshita

**Affiliations:** ^1^ The Japan Drug Information Institute in Pregnancy, Integrated Center for Women's Health National Center for Child Health and Development Tokyo Japan; ^2^ Department of Pharmacy National Center for Child Health and Development Tokyo Japan; ^3^ Aoki Obstetrics and Gynecology Clinic Tokyo Japan; ^4^ Tokyo Mother's Clinic Tokyo Japan; ^5^ Medical Affairs Department Maruho Co. Ltd. Osaka Japan

**Keywords:** amenamevir, breastfeeding, patient–initiated therapy (PIT), recurrent herpes simplex (RHS), relative infant dose (RID)

## Abstract

Patient–initiated therapy (PIT) is a treatment approach in which patients with recurrent herpes simplex self–administer medication at the onset of prodromal symptoms. PIT, which allows patients to receive medication in advance and initiate treatment at any time, is a therapeutic approach that alleviates the burden of urgent clinic visits for acute diseases like herpes simplex and benefits many people. Furthermore, its utility is particularly high for breastfeeding women for whom clinic visits can sometimes be difficult. When PIT is used in breastfeeding patients, potential drug transfer into breast milk and infant exposure must be considered. However, no studies have evaluated the extent of amenamevir transfer into human breast milk or its safety in infants. In this study, the relative infant dose (RID) was estimated from breast milk concentrations after a single oral dose of amenamevir. In the two cases evaluated, the estimated RID values were below 1%. This preliminary data suggest low estimated transfer of amenamevir into human milk after PIT. However, because breastfeeding was interrupted and infant exposure was not evaluated, clinical safety in breastfed infants remains undetermined.

**Trial Registration:** Japan Registry of Clinical Trials identifier: jRCTs031230591

## 1 Introduction

Recurrent herpes simplex (RHS) results from reactivation of latent herpes simplex virus, in sensory ganglia, typically triggered by immunocompromise, fatigue, UV exposure, or other factors [1]. In the clinical treatment of RHS, several oral antiviral drugs for herpes are available in Japan. According to the Drugs and Lactation Database, provided by the U.S. National Institutes of Health, both acyclovir and valacyclovir are considered safe for use during lactation [2]. Amenamevir and famciclovir have been approved for RHS treatment as PIT, which allows patients to start medication at their own discretion immediately upon recognizing early symptoms. The PIT strategy suppresses viral replication early by exposing the virus to high concentrations of the antiviral agent [3, 4, 5]. For breastfeeding women, seeing a medical professional immediately after symptom onset can be difficult due to challenges in bringing the infant or the practical impossibility of arranging childcare at short notice. In such cases, PIT can be a useful option for women with RHS. However, data to inform treatment decisions on amenamevir use during breastfeeding are lacking. Therefore, this study evaluated amenamevir transfer into breast milk.

## 2 Methods

This study was a single–arm, open–label, interventional trial led by the principal investigator from the National Center for Child Health and Development (NCCHD) and sub–investigators from four registered hospitals. The inclusion criteria included patients with RHS who were breastfeeding within 1 year after childbirth and able to suspend breastfeeding for 48 h after taking amenamevir. Informed consent was obtained from eligible patients meeting all inclusion criteria [6]. Patients were prescribed 1200 mg of amenamevir and instructed to carry it. They took the tablets after a meal within 6 h of recognizing prodromal symptoms [7]. Milk samples (100–150 mL) were collected at the mandatory time points of 4, 8, 24, 36, and 48–hours, as well as at optional time points after dosing, and 3 mL aliquots of each sample were stored under refrigeration for analysis. Subsequently, they visited the hospital to submit breast milk samples and records. During the visit, a single blood sample was collected to confirm the plasma concentration of amenamevir and ensure proper administration. The submitted samples were stored at −80°C or below until analysis. Amenamevir concentrations were measured by tandem mass spectrometry (LC–MS/MS) at Maruho Co. Ltd., Kyoto R&D Center [6]. The key validation parameters are 94.2%–105.4% for accuracy and 3.4%–6.1% for precision. Sample is stable for 7 days at 5°C and for 90 days at −30°C and −80°C. The primary outcome was the relative infant dose (RID) of amenamevir, calculated from the average drug concentration (C_ave [AUC(0–24h)]_), derived from AUC_0–24h_ estimated using the linear–log trapezoidal method. RID was defined as follows [8].

$$\mathrm{RID}\;\left(\%\right)=\frac{\text{theoretical infant daily dose}\;\left(\mathrm{mg}/\mathrm{kg}/\mathrm{day}\right)}{\text{maternal medication dose}\;\left(\mathrm{mg}/\mathrm{kg}/\mathrm{day}\right)}\times 100$$

Theoretical infant daily dose was defined as the drug concentration in breast milk (mg/mL) multiplied by the infant's milk intake (mL/kg/day) [9]. In this study, the infant's milk intake was set at 150 mL/kg/day [10]. RID_max_ based on the observed maximum concentration, representing a highly conservative theoretical estimate assuming continuous exposure at that level, was also evaluated as a secondary endpoint. The above RID calculations and various pharmacokinetic parameter calculations were performed by the NCCHD Pharmacy Department. Methodological details are described in the study protocol [6]. Infant adverse events were not assessed because breastfeeding was interrupted, and infant exposure did not occur.

## 3 Results

### 3.1 Patient Characteristics and Analysis Population

Twelve patients were enrolled in this study, of which two experienced RHS during the observation period. Both patients took amenamevir after a meal within 6 h of the onset of prodromal symptoms. The clinical diagnoses were herpes labialis for Case 1 and genital herpes for Case 2 (Table 1). The number of days from postpartum to recurrence was 105 days for Case 1 and 202 days for Case 2. Case 1 had a comorbidity of hypothyroidism. Case 2 completed breast milk collection at all five required time points as well as two optional time points. In contrast, Case 1 provided samples at only three of the five scheduled time points because two scheduled samples were not collected. Consequently, both patients were included in the Safety Analysis Set and Full Analysis Set, while only one patient met all the protocol requirements and was included in the Per–Protocol Set (Figure S1).

**TABLE 1:** Background characteristics of the study participants (FAS).

	Case 1	Case 2
Age	35 years	28 years
Weight	60.6 kg	53.1 kg
Clinical presentation	Recurrent herpes labialis	Recurrent genital herpes
Comorbidities	Hypothyroidism	None
Concomitant medications	None	None
Postnatal days	105	202

### 3.2 Drug Concentration in Breast Milk and Relative Infant Dose

The concentrations of amenamevir in breast milk at each time point are shown in Figure 1. The AUC_0–24h_ and C_ave [AUC(0–24h)]_ for Case 2 were 12200 ng·h/mL and 508.3 ng/mL, respectively. Because only one time point was available within 24 h post‐dose for Case 1, AUC_0–24h_ could not be calculated. The observed maximum concentration for Case 1 and Case 2 was 400 ng/mL at 3.5 h and 1390 ng/mL at 4.17 h, respectively. The RID, calculated using the primary endpoint C_ave [AUC(0–24h)]_, was 0.337% for Case 2. The RID_max_, calculated using observed maximum concentration, was 0.303% and 0.923%, respectively (Table 2).

**FIGURE 1:**
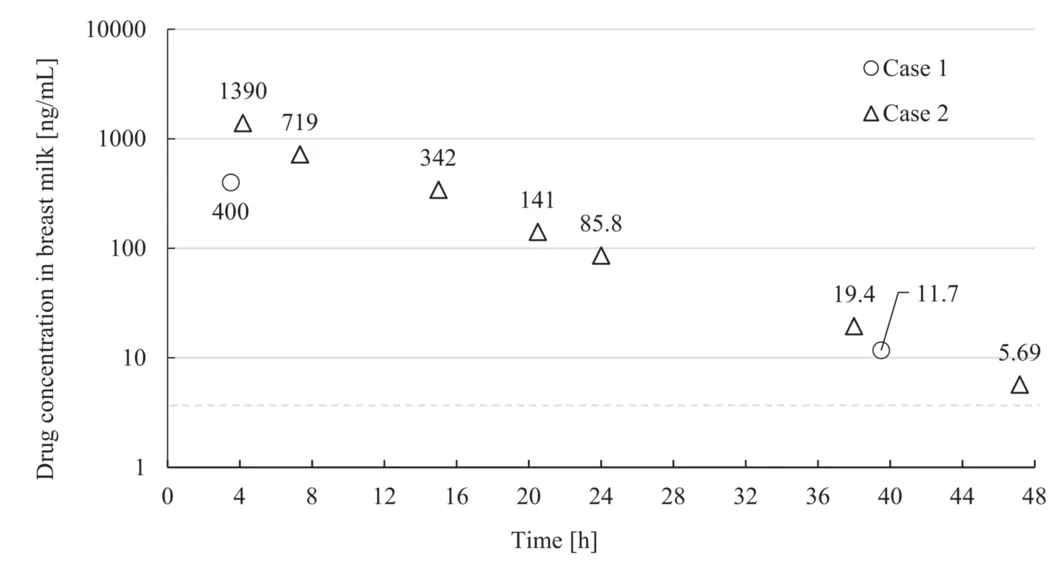
Concentration of amenamevir in breast milk over time after dosing. Breast milk concentrations were measured at 3.5, 39.5, and 51.8 h post–dose for Case 1, and at 4.17, 7.33, 15.0, 20.5, 24.0, 38.0, and 47.2 h post–dose for Case 2, as indicated by open circles for Case 1 and open triangles for Case 2. Notably, the concentration at the 51.8 h for Case 1 was below the lower limit of quantification (5.00 ng/mL), as indicated by the dashed horizontal line.

**TABLE 2:** RID, TID, and pharmacokinetic parameters.

	AUC_0–24h_ (ng h/mL)	C_ave [AUC (0–24h)]_ (ng/mL)	RID (%)	TID (mg/kg/day)	Observed maximum concentration (ng/mL)	RID_max_ (%)	TID_max_ (mg/kg/day)
Case 1	—	—	—	—	400	0.303	0.060
Case 2	12200	508.3	0.337	0.076	1390	0.923	0.209

*Note:* For Case 2, AUC_0–24h_ was calculated using the linear–log trapezoidal method. AUC_0–4h_ was estimated using a linear approximation (*f*(*x*) = 332.23*x* + 5.69), and AUC_4–24h_ was estimated by log‐linear extrapolation (*f*(*x*) = 2004.9e^−0.125*x*^), where C_4h_ was substituted with the concentration at 4.17 h.

Abbreviations: C_ave [AUC (0–24h)]_, the average drug concentration in breast milk from 0 to 24 h after amenamevir administration; Observed maximum concentration, the concentration observed at 3.5 h after administration in Case 1, and at 4.17 h after administration in Case 2; RID, the relative infant dose (RID) calculated using C_ave [AUC (0–24h)]_; RID_max_, the RID calculated using observed maximum concentration; TID, the theoretical infant dose (TID) calculated using C_ave [AUC (0–24h)]_; TID_max_, the TID calculated using observed maximum concentration.

### 3.3 Other Evaluation Results

The plasma concentrations of amenamevir were 876 ng/mL at 19.2 h post–dose in Case 1 and 399 ng/mL at 18.3 h post–dose in Case 2. These values did not show deviation from clinical trial data [7], indicating appropriate drug administration. No maternal adverse events were observed in either patient.

## 4 Discussion

We report the individual RID from the two evaluated cases. Because only limited milk sampling was available for Case 1, C_ave [AUC(0–24h)]_–based RID was calculated only for Case 2 and was 0.337%. Based on the maximum concentration observed in breast milk, the RID_max_ was 0.303% for Case 1 and 0.923% for Case 2. The maximum concentration in breast milk was observed at 3.5 h for Case 1 and 4.17 h for Case 2, both within the reported plasma T_max_ range (3.83 ± 0.41 h) [7]. Therefore, the RID_max_ values based on the observed maximum concentration in breast milk likely represent highly conservative estimates. All values were below the 10% RID threshold, which is widely recognized as one of the criteria for assessing potential effects on infants [2].

Although the study planned to enroll 20 patients and obtain five evaluable cases with recurrence [6], only 12 patients were enrolled, and recurrence was observed in only two patients during the study period. A substantial number of eligible patients declined participation due to the requirement to suspend breastfeeding for 48 h after dosing, which limited enrollment. In addition, the observed recurrence rate was approximately 0.6 episodes per person–year, lower than the rate of at least 1 episode per person–year expected based on the study protocol inclusion criteria [6]. These findings suggest that achieving the target sample size may require modification of the breastfeeding suspension period or extension of the observation period. Although the breastfeeding interruption period was set at 48 h based on the plasma elimination half‐life, amenamevir remained detectable in breast milk at 48 h after administration in Case 2. However, the measured concentration was very low.

Breastfeeding provides substantial benefits to infants, and maintaining maternal health is essential to support this process. However, the ethical justification for exposing infants to risks from medication is a primary concern, as they receive no direct therapeutic benefit. Therefore, research is only considered permissible if the potential benefits–to the mother or for future scientific understanding–decisively outweigh the risks to the infant. These ethical dilemmas have historically resulted in the exclusion of lactating women from most clinical trials. This has led to a critical “information gap,” with insufficient data on the safety of many drugs during breastfeeding. As a result, clinicians and mothers are often left to make challenging decisions about treatment with inadequate guidance [11]. We conducted this study to address these issues.

In conclusion, although the clinical safety of breastfeeding during amenamevir treatment remains undetermined because infant exposure has not been evaluated, this study provides rare human data on amenamevir transfer into breast milk.

## Funding

Maruho Co. Ltd. provided the necessary funding for this study. The company was also involved in the design of research plans, the design of statistical analyses, and the implementation of measurements using milk and plasma samples but was not directly involved in the implementation of data management. Furthermore, test kits for milk collection and, where necessary, breast pumps were provided to patients free of charge.

## Ethics Statement

This study was conducted in accordance with the ethical principles of the Declaration of Helsinki and fully complied with Japanese clinical trial regulations. The study was approved by the Certified Review Board of the NCCHD (CRB3200005).

## Conflicts of Interest

Sachi Koinuma, Mikako Goto, Jumpei Saito, Hiroaki Aoki, and Satoshi Hayashi declare that they have no financial or other competing interests related to the overall trial or any of the participating study sites. This investigation was performed as an industry–initiated collaborative clinical research project, and co–author Tomoyoshi Takeshita is an employee of Maruho Co. Ltd.

## Supporting information


**Figure S1:** Patient disposition.

## Data Availability

The data that support the findings of this study are available on request from the corresponding author. The data are not publicly available due to privacy or ethical restrictions.
