# Supplementary material for: Amenamevir Transfer into Breast Milk in Patients with Herpes Simplex Virus Infection: A Brief Report of a Prospective Multicenter Study
Source: J Dermatol. 2026 Aug 31;53(11):1682–5. doi: 10.1111/1346-8138.70474 (PMC13737436; doi:10.1111/1346-8138.70474)
Supplement: Supplementary file 1 [file JDE-53-1682-s001.docx]

Registration, n = 12

Full Analysis Set, n = 2

Per Protocol Set, n = 1

Safety Analysis Set, n = 2

Excluded, n = 1

∙ Missing milk sampling at one or more scheduled time points (4h, 8h, 24h, 36h, or 48h), n = 1

Excluded, n = 0

∙ No data obtained after initiation of protocol treatment, n = 0

∙ Later found to violate eligibility criteria, n = 0

Excluded, n = 10

∙ Did not receive any protocol treatment n=10
